# Is Positive Affect as a Trait Related to Higher Heart Rate Variability in a Stressful Situation?

**DOI:** 10.3390/ijerph20206919

**Published:** 2023-10-13

**Authors:** Sigrún Þóra Sveinsdóttir, Kamilla Rún Jóhannsdóttir

**Affiliations:** Department of Psychology, Reykjavik University, Menntavegur 1, 102 Reykjavik, Iceland; kamilla@ru.is

**Keywords:** heart rate, positive affect, parasympathetic activity, stress, emotional regulation

## Abstract

Most of the studies on the effect of trait positive affect (PA) and cardiovascular activity have focused on heart rate (HR) and blood pressure (BP) rather than heart rate variability (HRV). However, trait PA might sustain homeostasis for the autonomic system (ANS) by reducing activity in the sympathetic system (SNS) and increasing the activity in the parasympathetic system (PNS). A common index for the PNS is the vagal tone measured indirectly through HRV. The present study assessed whether trait PA influences cardiovascular response to various stress tasks by monitoring participants’ HRV measured by RMSSD (root mean square of successive differences) along with HR and interbeat interval (IBI). A total of 54 participants performed various cognitive tasks and Trier Social Stress Tasks while their vital signs were monitored, and trait PA was measured with PANAS. The cognitive tasks included both high- and low-stress tasks, including fatigue-inducing 20 min Stroop tasks. The results showed overall higher HRV as measured by RMSSD for participants who have higher levels of trait PA, indicating more PNS activity compared with low-trait-PA individuals, particularly at the end of the task performance during the fatigue induction. High-trait-PA individuals also had a lower HR during the fatigue-inducing task and a higher IBI. The results support previous work by further indicating a more adaptive response and consequently better emotional regulation for high-trait-PA individuals in a complex, prolonged task setting.

## 1. Introduction

There is growing evidence that the benefit of positive affect (PA) goes beyond the momentary experience of feeling good. According to several literature reviews, PA is associated with various health outcomes such as longer life [1,2,3], enhanced cardiovascular health [4], improved immune system/increased resistance to infection, and even better prognosis for diseases such as cancer, HIV, and diabetes [5,6,7]. The effects of PA on health are independent of negative affect (NA) [8,9], indicating that PA and NA are separate dimensions rather than bipolar opposites of the same scale [10]. 

The accumulating research literature on the influence of PA has inspired research to explore which biological mechanisms are associated with PA and lead to better physical health. Many of these studies have focused on cardiovascular reactivity and recovery [11,12,13,14]. Both high cardiovascular reactivity [15,16,17] and/or delayed recovery [18,19,20] are risk factors for cardiovascular diseases, and changes in emotions reflect changes in cardiovascular activity through the sympathetic nervous system (SNS) [21]. In contrast, faster cardiovascular recovery and lower reactivity indicate protective factors and are associated with activity in the parasympathetic nervous system (PNS) [22,23]. 

The results from studies on the associations of PA and cardiovascular activity examined after mood induction are mixed. In a sequence of studies by Fredrickson and Levenson (1998) and Tugade and Fredrickson (2004), they compared IBI (interbeat interval), aggregated finger pulse amplitude, HR, diastolic blood pressure (DBP), and systolic blood pressure (SBP) in university students who watched films made to induce PA and NA [11,24]. They found that recovery was quicker after viewing an amusing film compared to a film made to induce sadness or fear. Other similar studies on mood induction have found no significant effect of PA [22,25]. Dockray and Steptoe (2010) suggested that PA might mediate the cardiovascular response to NA or stress situations [26]. This is aligned with results from studies that have shown that PA is associated with quicker/faster recovery after a negative or stressful induction [11,12]. This indicates that positive emotions might influence cardiovascular activity through parasympathetic activation, which slows down the effects of the SNS [27].

Papousek et al. (2010) measured both trait and state PA and their influence on cardiovascular recovery after a simulated academic stress challenge [13]. They measured HR, HRV, and BP and found that after exposure to academic stress, participants with high levels of trait PA had more complete subjective and cardiovascular recovery compared to participants low in trait PA. More specifically, they found that high-trait-PA individuals had better DBP recovery and HRV (measured by LF/HF and LF) recovery. Trait PA was not related to recovery in SBP, HR, and HRV measured as HF (high frequency) [13]. Moreover, PA state was not correlated with cardiovascular recovery. Similarly, both Bostock et al. (2011) and Papousek et al. (2010) found that trait PA was associated with more efficient DBP recovery following stressful tasks and not with SBP and HR recovery or reactivity [13,28]. 

Pressman and Cohen (2005) suggested that trait PA might sustain homeostasis for the autonomic nervous system (ANS) by reducing activity in the SNS and increasing activity in the PNS [10]. A common index for the PNS is the vagal tone, which represents the activity of the vagus nerve, which is a vital component of the PNS. Vagal tone is measured indirectly through HRV with measures such as RMSSD (root mean square of successive differences) and HF [28,29,30,31]. As the vagus nerve influences the sinoartial node of the heart and therefore substantially influences HR, it is important to note that HRV is influenced by other factors such as respiration, hormonal levels, and blood pressure [32]. HRV refers to the variation over time between successive heartbeats. It is a useful signal to assess the activity of the ANS. The normal variation between heartbeats is caused by the autonomic regulation of the heart and the cardiovascular system. The balance between the SNS and PNS physiologically controls the HR, where an increase in the SNS causes HR acceleration and PNS deceleration [33]. In a review of HRV in psychology, Pham et al. (2021) reported that a high HRV is considered an indicator of a healthy regulatory system that can adapt successfully to environmental and psychological changes. Whereas low HRV is associated with poor cardiovascular health, cardiovascular diseases, as well as mental and cognitive issues [34]. 

Accumulating studies have found an association between HRV and positive emotions, indicating that high HRV is associated with positive emotions [35,36,37,38,39,40]. Oveis et al. (2009) found a stable positive association between basal vagal tone and trait PA [39]. Similarly, Wang et al. (2013) found a stable association between trait PA and HRV, independent of trait NA [37]. Interestingly, Schwerdtfeger and Gerteis (2014) found that state PA was associated with lower HRV and trait PA with higher HRV measured in everyday life [36]. 

The association between trait PA and HRV might be accounted for by the neurovisceral integration model, which hypothesizes that the overlap of brain structures implicated in cognitive, affective, and autonomic regulation explains the complicated interaction between these systems [27,41]. The cardiac vagal tone measured by HRV serves as an indicator of the performance of these systems. A higher resting HRV indicates better performance, more adaptive functioning, and self-regulation, which in turn contributes to more efficient emotional regulation. Lower resting HRV levels, in contrast, reflect poorer performance, less adaptive functioning, and self-regulation that leads to emotional dysregulation [42]. This relationship has been supported by studies, which have found a relationship between higher levels of HRV and better emotional regulation [43,44,45,46]. Furthermore, research has found that emotional regulation mediates the positive association between HRV and PA [47]. This, however, remains to be explored and supported further by research. Studies on trait PA have generally not found any connection between PA and HR [9,12,13,28]. It should be noted, however, that most of them have used fairly brief, heterogeneous stress tasks. In a study by Steptoe et al. (2005) they found that high trait PA in men was linked to overall lower HR during the day [48]. Furthermore, a very limited number of studies have examined the potential link between PA and HRV, particularly through the RMSSD measure. 

The current study aimed to further examine the association between trait PA and the cardiovascular response to a stressor by (a) looking at HRV measured with RMSSD as an indication of the activity of the PNS along with HR and IBI and (b) having participants perform on multiple tasks, both cognitive and social stressors, along with a fatigue-inducing 20 min Stroop task. Prior work has generally looked at the cardiovascular response with trait PA by using brief and often highly stressful tasks (e.g., public speaking). By including both types (cognitive and social stressors), as well as increasing the performance time and including a fatigue-inducing Stroop task, we increase the likelihood that the stressors reflect demands from natural settings and that they induce a reliably sufficient physiological response. This, in turn, can help to indicate whether the physiological activity can be generalized to a response to a real-world stressor. In addition, the setup allowed us to explore the development of the cardiovascular response over time as participants performed the various tasks for a total time of 66 min, where they performed continuously on the Stroop task for 20 min. It has been proposed that trait PA is associated with better adaptability to various task demands and cognitive flexibility [49,50] (e.g., Dreisbach & Goschke, 2004; Tugade & Fredrickson, 2004), but this has not been put to the test in a prolonged-task situation. A recovery period was included following the Trier stress task. After the recovery period, the participants performed the Stroop task for 20 min and then the PVT for 10 min. The subjective experience of stress and task demand was also measured using the NASA TLX questionnaire. As Cavanagh and Larkin (2018) emphasize the importance of including individual characteristics that might affect cardiovascular outcomes, so we controlled for sex and age in our analyses along with NA [51]. 

The first and main goal of the study was to assess whether PA as a trait influences the PNS during prolonged and varied task performance, as measured by the difference in HRV-RMSSD. A secondary goal was to look at the association between trait PA and cardiovascular response more generally by including HR and IBI (the bases for calculating any HRV measure). We expected participants who have high levels of trait PA to have a more enhanced PNS response to the stressor than those with a low levels of trait PA and that this effect would be independent of sex, age, and NA. Specifically, a more enhanced PNS response should be evident with a higher HRV-RMSSD. Although prior work has not found a clear connection between trait PA and HR, with the altered set-up in the present study, we expect to see HR and IBI differences with trait PA, particularly during the final stages of task performance with the 20 min Stroop task and the 10 min PVT. Given that trait PA enhances overall task adaptability and cognitive flexibility, high-trait-PA participants should be better at regulating their cardiovascular response during prolonged task performance.

## 2. Materials and Methods

### 2.1. Participants

A total of 54 individuals participated in the current study, 25 men (46%) and 29 women (53%). A power analysis was conducted with G*Power 3 software version 3.1.9.7 [52]. It revealed that 45 participants were needed to obtain a medium effect size (Cohen’s D = 0.5) (given α = 0.05). The participants were 19–50 years old (*MD =* 27.734 *SD =* 7.41), and they volunteered to be a part of the study through an advertisement. The advertisement was hung up in public areas such as universities, student lodging, swimming pools, and gyms. The advertisement was also electronically sent to students at Reykjavik University. Additionally, the advertisement was posted in private groups on Facebook. 

The study received approval from the National Bioethics Committee (VSN-21-087) and complied with its ethical standards. The participants read and signed a consent form before participating. They were encouraged to approach or contact the researchers if they experienced feelings of discomfort during the study. The participants had the right to withdraw their participation at any point during the study. Participants received a 4000 ISK gift card for taking part in the study. 

### 2.2. Materials 

#### 2.2.1. PANAS

The positive and negative affect schedule (PANAS) is a subjective assessment scale of positive (PA) and negative (NA) emotions [53]. There is a low correlation between PA, and they are measured as independent variables in factor analyses [54]. The assessment evaluates 20 emotions, 10 of them refer to positive emotions and 10 refer to negative emotions. The answers are on a five-point Likert scale and range from “very slightly or not at all” to “extremely” and refer to how much a particular emotion relates to the participant’s emotional state. The PANAS can be used to assess the current emotional state, the emotional state of the past month, or the past two weeks. In the current study, the assessment refers to the participant’s emotional state for the past two weeks. The assessment scale has been translated into Icelandic by Árni Halldórsson and Daníel Þór Ólason and is called the PANAS-IS [55]. It has strong internal reliability, corresponding with the reliability of the original PANAS [55,56]. The total score for both the PA and NA scales ranges from 10 to 50. A higher score on the PA scale indicates more PA and a higher score on the NA scale indicates more NA. 

#### 2.2.2. Cognitive Tests

The Balloon Analogue Risk Task (BART) consists of a sequence of decisions in uncertainty. In the task, participants are asked to press a button to pump air into a virtual balloon [57]. With every press, the balloon inflates, and participants can see an increase in their earned money reward. The balloon can explode at any time, and if it does, the earned money reward is lost. With each pump, participants must decide if they want to cash out the already earned money or pump again and risk the balloon exploding and losing their earned reward. The participants do not know when the balloon will explode, thus the decision is always made under uncertainty. The BART requires participants to balance their desire for a higher reward with their desire to avoid the loss of reward under uncertainty. The participants can collect a monetary reward or leave the risky circumstances whenever, much like in real-world risky decision making. Therefore, the BART has high validity [58,59].

The Bell Test measures visual selective and focused attention, visual perception, and visual-motor-processing speed [60]. In the test, participants locate and cross out 35 bells, which are mixed with another 315 distracting figures on a horizontal sheet of paper. Participants complete the task in the shortest possible time, and their completion time is recorded. The test is scored by the number of correctly crossed-out bells and the completion time. The highest score possible is 35, and the completion time is always under 5 min. Studies have demonstrated the test’s validity in identifying patients suffering from a stroke and brain damage in the right cerebral hemisphere [61,62,63].

The Corsi Block-Tapping Test is a short-term memory test similar to a digit span test. It is named after its creator, Philip Michael Corsi, who developed it as a doctorate student [64]. The Corsi test assesses short-term vision and spatial memory. The test was not originally developed as a computer test. The test is normally nine squared blocks placed on a wooden table, but several digital versions have been developed [65]. In the test, the participant sits in front of a computer screen and watches squared blocks light up, one by one, in a certain order. The participant is then supposed to click the blocks in the same order as they lit up. 

The Judgment of Line and Position Task (JLAP) is a cognitive task that assesses visuospatial ability [66]. It is adapted from Collaer (2001), where the target line is displayed above a reference array [67]. The participant’s goal is to match the target line to the relevant stimuli line in the reference array. They are presented with 20 lines with various accuracy scores that scope from 0 to 20, and they have 10 s to match it with the reference array [66]. 

The Psychomotor Vigilance Task (PVT) was originally developed in 1985 to measure sustained attention [68]. Since then, many studies have demonstrated the task’s acuity, and it is one of the most used neurocognitive tests [69,70]. In the task, the participant is asked to press a button as quickly as possible when a red square appears on a computer screen. The task is 10 min long and measures response time and sustained attention [68]. The PVT is believed to reflect the participant’s state of attention and performance. It has been connected to a lack of sleep and fatigue [70]. The PVT has been evaluated as a reliable measure of attention and vigilance [71]. 

The Stroop test (ST) is an attention test that is a popular neuropsychological measuring device designed by Stroop [72]. It is easy to use and has shown a significant correlation with other, more complex, psychological measuring instruments [73]. The Stroop test has been used to measure various mental abilities, such as the cognitive control of automatic processes and selective attention [74]. The test consists of two situations, firstly, a word appears in a color that is inconsistent with the meaning of the word (e.g., the word “blue” has a red color), and secondly, a word appears in a color that corresponds to the meaning of the word (e.g., the word “green” is green). The participant’s goal is to ignore the meaning of the word (do not read the word) and name the color of the word that appears. Three levels of Stroop were used in the present study: 100% congruent (color and color name match), partly incongruent (30 and 70% of the color names match their color), and time-limited (each word presented for a limited time) [75]. The three levels were presented in a random order and repeatedly for a total of 20 min.

The Trail Making Test (TMT) consists of two parts: A and B [76,77]. In TMT-A, participants draw a line between encircled numbers in the correct order as fast as they can, while their completion time is recorded [78]. In TMT-B, participants draw a line between encircled numbers and letters, alternating between the two as fast as they can, while their completion time is recorded. TMT-A measures visual search and processing speed, and TMT-B is believed to measure executive function. The TMT is one of the most used neuropsychological tests, and it is believed to have strong reliability [79].

A verbal fluency task evaluates the spontaneous recall of words, either words that start with a specific letter (letter fluency) or words that belong to a specific group such as animals (category fluency). The test is believed to measure control and semantic verbal skills [80,81]. Verbal fluency was assessed using a short version of the COWAT translated to Icelandic, using the letters H and S [82]. Even though it is more common to use a version that uses three letters, a two-letter version is often used to shorten the testing time [83,84]. Participants are asked to name as many words as they can starting with a specific letter in one minute. The number of words listed (letter fluency for letters H and S) and the mean number of words (mean letter fluency) are recorded. Category fluency is assessed by asking participants to name as many animals as they can in one minute [85]. The test was translated and adapted by Dr. Brynja B. Magnusdottir [86].

#### 2.2.3. VAS

The visual analogue scale (VAS) is a measuring tool used to assess, e.g., perceived stress, which is believed to be a dimensionless or continuous value, i.e., something that is challenging to measure directly [87]. The scale is often used in epidemiological and clinical research to measure the strength or frequency of symptoms [88]. The current study used a visual analog scale to assess subjective stress and fatigue. It was assessed on a line that was about 120 mm, so the participants could mark the line anywhere between 0 mm and 120 mm [89]. The questions were (1) indicate how much stress you are experiencing right now by putting a mark through the line here below; (2) indicate how much fatigue you are experiencing right now by putting a mark through the line here below; and (3) indicate how focused you are right now by putting a mark through the line here below. 

#### 2.2.4. NASA-TLX Questionnaire

A modified version of the NASA Task Load Index (TLX) questionnaire was used to assess the subjective experience of workload, which is called the Raw-TLX questionnaire [90]. In the Raw-TLX version, the rating scales are not weighted. Rather, an average score for each of the rating scales is calculated. Research has shown that the Raw-TLX version has similar sensitivity to the original questionnaire but is simpler in terms of its implementation [90]. Additionally, the present study used five of the original six NASA-TLX rating scales (dimensions); mental demand, temporal demand, performance, effort, and frustration. Each of the rating scales had a 20-step bipolar scale that had “very low” on one end and “very high“ on the other. Every rating scale gave a maximum of 10 points (1 point for a pair of steps on the 20-step scale). In the present study, the subscales were analyzed separately rather than calculating a single workload score. The NASA-TLX scale is a reliable measure of subjective workload [91,92,93]. The internal validity for the five dimensions (mental demand, temporal demand, performance, effort, and frustration) used in the present study was fairly high, with Cronbach ‘s alpha ranging from *α* = 0.796 to 0.896. 

#### 2.2.5. Stress Test

The Trier Social Stress Test (TSST) is used to examine stress responses in a laboratory setting using a modified version [94]. The participants are asked to imagine that they have been invited to a job interview for their dream summer job. They are asked to explain why they are the right person for the job in front of a recording camera. They are told that the researcher and three people on a job interview committee will later watch and evaluate their speech. The participants are given three minutes to prepare their speech. While preparing their speech they are allowed to write down key points but are not allowed to look at their key points during the presentation. After the three-minute preparation, the participants are informed that they will have four minutes to present their speech and that they will have to talk through the four minutes. If the participants become silent before the four minutes pass, they are asked to continue. When the four minutes pass, the participants are told that the job requires mathematical skills and that theirs will have to be evaluated. The participants are asked to subtract 17 from 2023 out loud as often as they can in three minutes. If they give a wrong answer, they must start again from 2023.

#### 2.2.6. Equipment

A medical Caretaker was used to monitor the participants’ vital signs while they performed the various cognitive and social stress tests [95]. The Caretaker provides vital signs, such as HR, IBI (e.g., changes between heartbeats), respiration rate, and blood oxygen level [95]. It is approved by the US Food and Drug Administration and is CE-cleared for the measurement of HR, respiratory rate, and self-calibration (FDA K163255). An upper arm cuff measurement of blood pressure was taken separately to calibrate the Caretaker device. It is a continuous noninvasive physiological monitor (Caretaker Medical LLC, Charlottesville, VA), and according to Kwon et al. (2022), “This device uses low pressure (~35–45 mmHG), pump-inflated finger cuff that pneumatically couples arterial pulsations via a pressure line to a custom-designed piezo-electric pressure sensor for detection and analysis.” [96] (pp. 1–2). Furthermore, the Caretaker has been estimated to fulfil the gold standard of invasively measured arterial BP as well as provide adequate IBI for HRV tracking compared to other non-ECG systems [96]. 

### 2.3. Procedure

**Figure 1:**
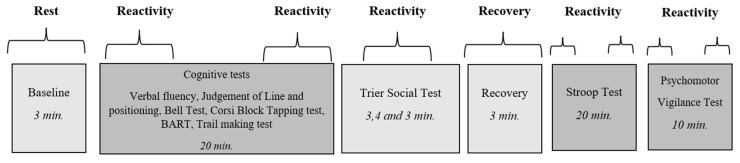
The experimental procedure. Participants were measured with a medical device, the Caretaker, throughout the whole experiment, starting with the baseline period, cognitive tests (BART = the Balloon Analogue Risk TASK), Trier Social Stress Test, recovery period, and ending with the Stroop and the PVTs. Reactivity means were calculated for different time segments throughout the experiment.

The experimental session was divided into three parts: cognitive task performance, the Trier Stress Test, and recovery, and finally, performing the Stroop task over time (20 min) and the PVT (10 min). Before starting the cognitive tests, the participants filled out the PANAS questionnaire. The Caretaker device was then put on the participants and their blood pressure was measured to calibrate the device. A 3 min baseline session was recorded where the participants were asked to relax and rest before the experiment, and the procedure can be seen in Figure 1. After baseline recordings, the participants performed several cognitive tests (e.g., Verbal fluency, Judgement of Line and Positioning, Bell Test, Corsi Block Tapping Test, BART, Trail Making Test). Participants were given standardized guidelines for the cognitive tests and, for some, a practice trial that was not included in the final scoring. Following the cognitive tests, participants were given instructions for the Trier Social Stress Test, where they had a three-minute preparation period for the preparation of the speech, four minutes for the speech, and finally, three minutes for the math test. A 3 min recovery period followed the Trier Stress Test. In the final part of the experiment, participants performed the Stroop test for 20 min with the three levels appearing repeatedly in random order. The VAS scale and NASA TLX were used to assess whether subjective stress, fatigue, and workload varied with PA. The first measure was taken at the end of the Trier Test and the other five at different time points during the continuous performance on the Stroop task. The experiment ended with participants performing the PVT for 10 min. 

### 2.4. Recording of HRV

Continuous measurements of IBI were taken with the medical device, the Caretaker [95]. The caretaker measures pressure changes in the participant’s finger and derives the HR and BP from the measurements. The cardiovascular data from 11 periods were analyzed in this study, and HRV was calculated in the time domain with the RMSSD (ms), the root mean square of successive differences of RR intervals. The following periods were analyzed: the baseline, the beginning and the end of the cognitive tasks, the preparation for the speech, the speech, a math problem, the recovery time, and the beginning and the end of both the Stroop and the PVT. The HR data were visually inspected in the preprocessing of the data, and a modest amount of noise and artifacts were detected. The main cause of the noise for some of the participants was likely due to movements by the participant’s finger starting in the Trier Social Stress Test, which created higher HRV values. An ARIMA model was created from the HR data from each participant to filter out the noise. A measurement was considered an error if its residual was over three standard deviations based on a residual distribution from all of the participants. For example, if the IBI was 700 and the ARIMA model estimated that the next IBI should be 740 but was 300, the residual was 440. If a measurement residual was outside the three standard deviations limit, it was linearly interpolated. However, most of the participants had an error rate under 1%, that is, 4 of the participants had an error rate over 10% and were therefore removed from the dataset. 

The HR dataset was processed by using an open-source Python package called pyhrv (https://pypi.org/project/pyhrv/) accessed on 1 November 2021. HRV can be assessed by many variables, and the choice is dependent upon the subject of interest and the research question itself. An HRV analysis can be conducted in the time domain, frequency domain, and non-linear indices [32]. In the time domain, there are the SDNN (standard deviation of all R–R intervals), RMSSD, pNN50 (percentage of successive normal sinus RR intervals greater than 50 ms), and peak-valley (time-domain filter dynamically centered at the exact ongoing respiratory frequency). All of them represent the vagal tone except for SDNN. The vagal tone is the vagus nerve activity that represents the PNS [32]. For the frequency domain, the analysis must filter the signal into different bands: ultra-low frequencies (ULFs), very low-frequencies (VLFs), low frequencies (LFs), high frequencies (HFs), and a low frequency/high-frequency ratio (LF/HF). Here, HF represents the vagal tone, LF and LF/HF are a mix of sympathetic and vagal activity, and ULF and VLF are other bodily functions such as body temperature and hormonal mechanisms. According to Laborde et al. (2017), the RMSSD gives a better estimate of vagal tone than the pNN50 and is relatively free from respiratory effects, unlike HFs. In the current study, the RMSSD was therefore chosen as the parameter for HRV [32].

### 2.5. Statistical Analysis

Statistical analyses were carried out by IBM SPSS Statistics 27. It revealed that 45 participants were needed to obtain a medium effect size (Cohen’s D = 0.5) (given α = 0.05). The average HRV-RMSSD, HR, and IBI were calculated for different time segments, with each segment lasting for 3–4 min. There were eleven time segments in total; the 3 min baseline, the first and last 4 min of the cognitive tasks (pre- and postcog), 3 min for the preparation of the speech (anticipation), 4 min for the speech (stress), 3 min for the math problems, a 3 min recovery time, the first and last 4 min of the Stroop test (pre- and poststroop), and the first and last 3 min of the PVT (pre- and post-PVT). The effect of PA was examined by a repeated-measure analysis of covariance ANCOVA, where time was the within-subject factor and PA the main predictor variable, with sex, age, and NA as covariates. Additional repeated measures were conducted for the Stroop task and PVT, where the time segments were divided into 4 segments for the Stroop task (5 min per segment) and 2 for PVT (5 min per segment). For the significance tests, the PA was entered into the model as a continuous variable. The HRV-RMSSD, HR, and IBI values were plotted for high- and low-PA individuals using a median split (median = 29) and standard error bars for visual inspection of the difference between the two groups for each time segment. Greenhouse–Geisser adjustments were used to fulfill the requirements of sphericity where needed. The potential quadratic relationships between PA, the predictor variable, and the HRV-RMSSD were examined, and they were non-significant. This suggests that the linear relationship between the two variables is probably a more appropriate representation of their association. Additionally, the Partial Eta-Squared, *ηp*^2^, was calculated, and according to Gray and Kinnear (2012), a small effect size is between 0.01 and 0.06, a medium effect size is between 0.06 and 0.14, and a large effect size is 0.14 or higher [97].

Pearson correlation was performed for a supplementary analysis of the relationship between PA and reactivity (HRV-RMSSD, HR, and IBI averages), followed by a linear regression if there was a significant correlation. An independent *t*-test was used to explore the further difference between high- and low-PA individuals on HR. To assess whether subjective stress and workload varied with PA, repeated-measure ANCOVA were conducted for the VAS and NASATLX questionnaire, with time as the within-subject variable (6 measures) and PA as the main predictor variable, independently for each of the five NASA TLX questions and the three VAS questions. The analyses controlled for sex, age, and NA.

## 3. Results

The mean age of the participants was 27.74 with a standard deviation of 7.416, and the mean score for PA was 28.46 with a standard deviation of 6.51, and 19.05 for NA with a standard deviation of 6.14. The study had 54 participants in total, 25 men and 29 women. However, eight of them had missing data and were excluded from the final analysis. Therefore, the final analysis was based on 46 participants, 21 men and 25 women. The participants were grouped based on their scores for positive affect (PA) on the PANAS. The low-PA group had a mean score of 22.96 with a standard deviation of 3.96, and the high-PA group had a mean score of 32.90 with a standard deviation of 4.43. The median split (PA = 29) was used for plotting the difference between high- and low-trait PA, resulting in 29 high-trait and 25 low-trait individuals. The PANAS had internal consistency (*α* = 0.724), and the positive affect subscale had high internal consistency (*α* = 0.859)

### 3.1. HRV and Trait PA

**Figure 2:**
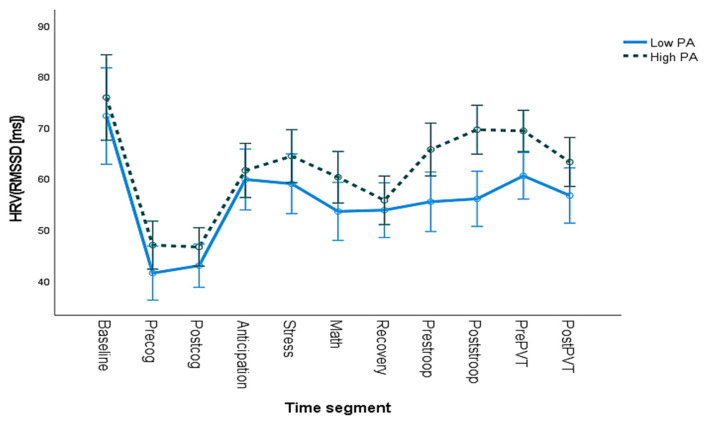
HRV (RMSSD) averages for different time segments of the experiment for high and low positive affect (PA) as a trait. The root-mean-square difference of successive normal R–R intervals (RMSSD) in milliseconds (ms) for each of the time segments (pre- and postcog = first and last 4 min of the cognitive tasks; pre- and poststroop = first and last 4 min of the Stroop task; pre- and postPVT = first and last 3 min of the PVT). Bars represent standard errors of the mean.

The results from the repeated-measures ANCOVA for the HRV-RMSSD showed a statistically significant main effect for PA (F_(1,41)_= 4.269, *p* < 0.05). Furthermore, the Partial Eta-Squared, *ηp^2^*, for the main effect was 0.094, indicating a medium effect size, and 0.018 for the interaction effect, indicating a small effect size. As can be seen in Figure 2, the high-PA individuals tended to have a higher HRV (indicating more PNS activity) compared to the low-PA individuals, and this difference is particularly noticeable in the second half of the study during the 20 min of continuous Stroop task performance and the PVT. Standard error bars show significantly different RMSSD values for high- and low-trait-PA individuals for the Stroop fatigue session and the first part of the PVT. A regression model showed that PA explained the variance of HRV poststroop (F_(1,46)_ = 3.910, *p* < 0.05, R^2^ = 0.078).

**Figure 3:**
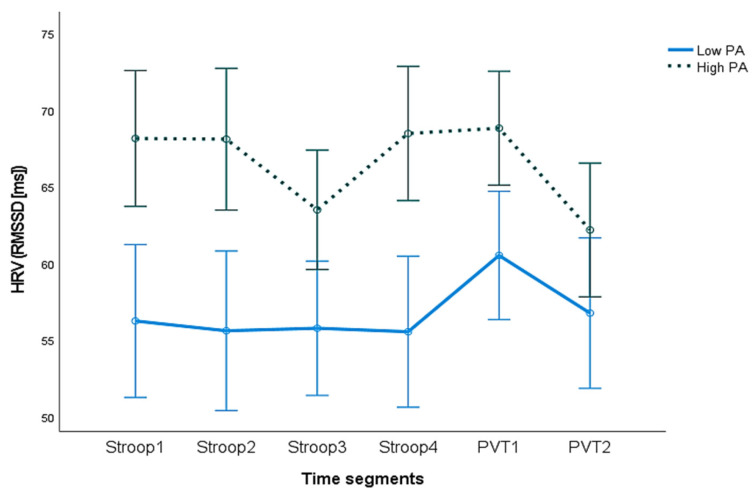
HRV (RMSSD) averages for the Stroop task and the Psychomotor Vigilance Task time segments of the experiment for high and low positive affect (PA) as a trait. The root-mean-square difference of successive normal R–R intervals (RMSSD) in milliseconds (ms) for each of the time segments (Stroop1-Stroop4 = 20 min of the task divided into 4 segments; PVT1-2 = 10 min of the task divided into 2 segments). Bars represent standard errors of the mean.

This difference was further assessed with a repeated-measures ANCOVA for the HRV-RMSSD, specifically for the Stroop task and the PVT. The test revealed a statistically significant main effect for PA (F_(1,46)_= 7.373, *p* < 0.05) when controlling for sex, age, baseline, and NA. The Partial Eta-Squared, *ηp*^2^, was 0.149 for the main effect, indicating a large effect size, and 0.026 for the interaction effect, indicating a small effect size. As can be seen in Figure 3, high-trait-PA individuals have a higher HRV compared to low-PA individuals throughout the 20 min in Stroop and PVT.

### 3.2. IBI, HR, and Trait PA

The repeated-measure ANCOVA for the IBI was not significant for the main or interaction effect. For the IBI, there was a negative correlation between PA and reactivity periods poststroop (r_(53)_= −0.287, *p* < 0.05) and prePVT (r_(53)_ = −0.304, *p* < 0.05). Moreover, a regression model showed that PA explained the variance of IBI reactivity for post-Stroop (F_(1,53)_ = 4.733 *p* < 0.05, R^2^ = 0.082) and prePVT (F_(1,53)_ = 4.707 *p* < 0.05, R_2_ = 0.082) periods. The Partial Eta-Squared, *ηp*^2^, was 0.014 for the main effect, indicating a large effect size, and 0.024 for the interaction effect, indicating a small effect size.

The high-trait-PA individuals had a higher IBI than the low-trait-PA individuals towards the end of the experimental set-up during the 20 min Stroop fatigue induction and the 10 min PVT at the end.

The repeated-measure ANCOVA for HR did not show a significant main or interaction effect. The independent *t*-tests revealed a significant difference between the groups in the post-Stroop (t_(50)_ = 2.266, *p* < 0.05) and the prePVT conditions (t_(53)_ = 2.041, *p* < 0.05). Additionally, there was a negative correlation between PA and the reactivity periods poststroop (r_(51)_ = −0.281, *p* < 0.05) and PrePVT (r_(51)_ = −0.310, *p* < 0.05). Further, a regression model showed that PA explained the variance of HR reactivity for the poststroop (F_(1,50)_ = 4.296 *p* < 0.05, R^2^ = 0.079) and PrePVT (F_(1,52)_ = 5.627 *p* < 0.05, R^2^ = 0.096) periods. The Partial Eta-Squared, *ηp*^2^, was 0.020 for both the main and interaction effects, indicating a small effect size.

### 3.3. Subjective Assessment of Stress and Workload

Additional ANCOVAs were carried out for the subjective stress assessments (VAS) and workload (NASA-TLX). There was a significant main effect of PA (F_(1,48)_ = 4.996, *p* = 0.030) on subjective stress. The Partial Eta-Squared, *ηp*^2^, was 0.094 for the main effect, indicating a medium effect size. There was also a significant main effect of PA on the experienced mental demand of the task (F_(1,48)_ = 5.152, *p* = 0.028), and for the experienced temporal demand F_(1,48)_ = 6.215, *p* = 0.016). The Partial Eta-Squared, *ηp*^2^, was 0.106 for the main effect of mental demand and 0.115 for that of temporal demand, indicating a medium effect size for both parameters. The main effect of PA on the experienced fatigue was close to being significant (F_(1,48)_ = 3.867, *p* = 0.055); furthermore, the Partial Eta-Squared, *ηp*^2^, was 0.075 for the main effect, indicating a medium effect size. No other main effect or interaction was significant. When controlling for sex, age, and NA, the higher-trait-PA individuals rated their experienced stress as well as the mental and temporal demand of the Trier task and the different segments of the 20 min Stroop task higher than the low-trait-PA individuals.

## 4. Discussion

This study aimed to assess to what extent HRV, measured as the RMSSD or the PNS activity, varied with trait PA during prolonged and varied task performance. It was predicted that participants who have high trait PA would have enhanced PNS activity compared with low-trait-PA individuals and that this effect would be independent of sex, age, and NA. This was tested by exposing participants to multiple tasks, both cognitive and social stressors, along with a fatigue-inducing 20 min Stroop task and a 10 min PVT at the end. In total, the experimental session took about an hour. A recovery period following the stress task was measured. The main results from this study were that the HRV-RMSSD averages varied for individuals with high and low trait PA. High-trait-PA individuals had higher HRV compared with low-trait-PA individuals, particularly for the latter part of the study, that is, after the stress task and during the part when participants performed the Stroop task continuously for 20 min and the PVT for 10 min. Additionally, high-trait-PA individuals rated the Trier task and the Stroop task as being more stressful and more mentally and temporally demanding compared with low-trait-PA individuals.

In addition to HRV, the study also examined the association between trait PA and both HR and IBI. Although most studies using brief stress tasks (e.g., social speaking) have not found a significant link between HR and trait PA, there is some indication that in real-life settings, trait PA may contribute to HR regulation [50]. Our assumption, therefore, was that in the current set-up, there might be a difference in how HR and similarly, IBI, are regulated between high- and low-trait-PA individuals, particularly during the fatigue induction at the end of the experimental set-up. The results from the present study are mainly in line with our expectations, as both HR and IBI were statistically different for high- and low-trait-PA during the final stages of the experiment, with high-trait-PA individuals having lower HRs and higher IBIs. HR has generally not been studied with trait PA for a prolonged time. This might explain the lack of association between HR and trait PA in studies within this field [9,12,13,31]. That is, the difference between HR in high- and low-trait-PA might be explained by task adaptability and cognitive flexibility during prolonged, challenging task situations [11,53]. There is considerable evidence for the beneficial effect of positive affect on cognitive flexibility [24,98,99]. That is, high-trait-PA individuals might cope better with a prolonged task situation compared to low-trait-PA individuals regarding cognitive capacity, and that might result in a lower HR at the end of the challenging situation.

### 4.1. Trait PA as a Buffer against Harmful Effects

The findings of this study provide indications that support the hypothesis that the dispositional levels of PA may serve as a buffer against the potentially harmful impact of stress on our biological systems [10]. This aligns with the stress-buffering model of PA, which posits that PA can influence stress and coping appraisals, potentially reducing cardiovascular reactivity and enhancing cardiovascular recovery [8]. This model resonates with earlier research by Barbara Fredrickson and her colleagues, who introduced the concept of the “undoing effect of positive affect”. According to this concept, positive emotions may have the capacity to mitigate or reverse the effects of negative emotions [11,100]. This effect has been studied extensively with various physiological signals, including the cardiovascular response, with many studies reporting stress-buffering effects associated with PA [11,35,40]. The findings of this study, while preliminary, contribute to this growing body of evidence, providing insight into these potential effects within the context of prolonged and diverse task performance settings.

### 4.2. HRV for PNS Activity and Trait PA

There are, to date, relatively few studies that have explored the relationship between PA and HRV [13,101]. Even fewer have studied the relationships between trait PA and HRV using the RMSSD measure. The results here showed a significantly higher HRV as measured by RMSSD for high-trait-PA individuals during the 20 min Stroop task fatigue induction as well as for most of the 10 min PVT performance at the end of the experimental set-up, indicating more parasympathetic activity for high-trait-PA individuals. The standard error bars did not distinguish between the high and low groups for the low-stress cognitive tasks and during the high-stress Trier social task. The results here are in line with previous research showing that PA is associated with more enhanced PNS activity, independent of NA. Kok et al. (2013) found a relationship between a positive state and the HRV-measured HF of the PNS, where an increase in positive emotion produced an increase in vagal tone [101]. Papousek et al. (2010) found that trait PA was related to enhanced recovery in HRV measured by the ratio LF/HF, considered to represent the balance between the SNS and the PNS, during a simulated academic stress task [13,32]. A considerable number of studies have also found an association between HRV and both state and trait PA when HRV is measured at baseline and in everyday life [39,41,102,103].

RMSSD is regarded as the most accurate measure of the vagal tone representing PNS activity and as being relatively free from respiratory effects [32]. Former studies that have assessed the connection between PA both as a trait and state and HRV recovery and reactivity have used measures such as HF or LF/HF [13,101]. This study is therefore one of the first to use the RMSSD in this context. Several studies have found that DBP and SBP vary with trait PA for different stressors [12,14,31], indicating less SNS activity for high-PA individuals. The results here add to the existing findings by further showing that trait PA is associated with enhanced PNS activity and that this may be even more pronounced during more demanding and prolonged task situations such as a fatigue-inducing 20 min Stroop task and 10 min PVT at the end of an hour-long task performance session.

### 4.3. HRV, PA, and Emotional Regulation

The experimental setup in the present study allowed us to examine the PNS activity with trait PA in demanding and prolonged task situations. Interestingly, the results showed that it was particularly at the end of the Stroop and PVT performances, when participants were probably getting tired and frustrated, that high-trait-PA individuals showed more PNS activity compared with low-trait-PA individuals. This indicates that the high-trait-PA individuals had more emotional regulation and therefore showed a more adaptive response in the demanding situation. In support of this idea, research has shown that increased activity in brain areas linked to trait PA is associated with improved automatic emotional regulation following an effective challenge and a greater capacity to self-regulate negative emotions [104,105]. Furthermore, it has been hypothesized that the relationships between affective flexibility and HRV can be seen as a complex interplay between autonomic, cognitive, and emotion regulation systems [30,41]. An interaction that the neurovisceral integration model explains is evident in the convergence of the brain structures for the central autonomic network and neural circuits behind cognitive and emotional regulation systems [30,106,107]. These systems in turn guide goal-directed behavior and facilitate flexible adaption toward diverse environmental demands. The neurovisceral integration model proposes that HRV can be utilized as an indicator of the performance of these cortico-subcortical neural circuits and that a higher baseline HRV indicates a more efficient performance of this network [106]. This idea has been supported by research that shows an association between cognitive, emotional, and physiological regulation and vagal tone measured by baseline HRV [30,107]. Additionally, accumulating research has demonstrated connections between effective emotional regulation and higher baseline HRV [43,44]. Interestingly, the measures of the subjective experience of stress and task demand showed that high-PA-trait individuals experienced more task demand and stress. One possibility could be that this indicates that they have more awareness and control over the situation, realizing that it is demanding and stressful and reacting accordingly. This, however, remains to be explored more thoroughly by research and might be explained in other ways.

The strength of this study is that it includes two types of stressors, social and cognitive tasks, which increases the likelihood of representing an ecologically valid setting. The results supported unequivocally that both cardiovascular and subjective stress were successfully induced for the participants. Additionally, few studies have observed the relationships between PA and cardiovascular response with a prolonged stress intervention. This, in turn, showed that participants who had high levels of trait PA had a higher RMSSD or more PNS activity at the final stage of the intervention than those who had low levels of trait PA. Indicating that participants who have high levels of trait PA have more tolerance for prolonged stress and a more adaptive response in the ANS, reflected in higher PNS activity during stressful conditions.

### 4.4. Limitations

This study has several limitations that can influence the interpretability of the results. Firstly, even though the assessment of HRV was carried out with a device that is estimated to be sufficient for providing IBI measures for HRV compared to other non-ECG systems, it is likely that the pump-inflated finger cuff leads to noise to a modest degree that can affect the accuracy of the data. It should, however, be noted that the noise was limited and addressed with the relevant data filters. Secondly, a limitation of this study is that the non-ECG measure that was used may not have captured the most accurate cardiac activity of the participants, such as an ECG measure does, which might affect the validity and reliability of the results. Thirdly, the participants were a group of relatively healthy university students representing a homogenous sample of the population, limiting the capability to generalize this study to other groups. Additionally, even though the measure of PA as a trait is well accepted and used in the research field, it is not an exhaustive list of positive emotions. The theoretical orientation behind the PANAS assumes that PA represents high-arousal emotions and pleasures such as being alert, excited, etc., and neglecting positive emotions such as calm, love, or happiness [8]. Furthermore, even though it was controlled for individual characteristics such as sex and age, it would have been optimal to control for other potential confounding variables such as resilience, flourishing, and appraisal style. As Cavanagh and Larkin (2018) point out, these factors have been hypothesized to be associated with the tendency to experience positive emotions [54].

## 5. Conclusions

To summarize, the finding of the present study was that PA as a trait influences cardiovascular response, as measured by differences in the HRV-RMSSD. The participants who had high levels of PA as a trait showed more enhanced PNS activity than those who had low levels of trait PA, independent of sex, age, and NA, during a prolonged task situation with multiple stressor tasks. Moreover, participants with high levels of trait PA showed less HR and higher IBI during the latter part of the experiment. The present findings, therefore, support prior research on the relationship between PA and cardiovascular activity, building on prior studies by exploring the relationships over a prolonged period of time. Furthermore, it contributes additional information about the relationship between PA as a trait and HRV.

## Data Availability

The data presented in this study are available on request from the corresponding author. The data are not publicly available currently due to storage on-site at Reykjavík University.
